# Growth in the Size of Peritoneal Dialysis Facilities: A Path Toward Higher Quality or a Missed Opportunity?

**DOI:** 10.34067/KID.0000001077

**Published:** 2026-01-29

**Authors:** Hania Kassem, Kevin F. Erickson

**Affiliations:** 1 Section of Nephrology, Baylor College of Medicine, Houston, Texas; 2 Baker Institute for Public Policy, Rice University, Houston, Texas

**Keywords:** economic analysis, health policy, peritoneal dialysis

Home dialysis rates in the United States remain consistently lower than those observed in other developed nations. Over the past decade, policy initiatives have aimed to increase the adoption of home dialysis, yielding mixed outcomes. Notably, the proportion of patients receiving home dialysis rose from 8.5% in 2012 to 14.5% in 2022.^1^ This increase has been largely attributed to the US Centers for Medicare and Medicaid Services (CMS) implementation of the Expanded Prospective Payment System in 2011, which introduced financial incentives for facilities offering home-based modalities. Conversely, the ESKD Treatment Choices model, another CMS initiative launched to promote home dialysis and kidney transplantation, did not produce a significant change in utilization rates.^2^

In the context of peritoneal dialysis (PD), efforts to expand its use have been accompanied by growing recognition of the potential impact of facility size on patient outcomes. PD programs with higher patient censuses experience lower rates of technique failure and peritonitis.^3^ One explanation may be that larger facilities are better equipped to handle technical difficulties and have more resources to address issues related to patient and carer burnout.

In this issue of Kidney360, Shah *et al*. examine whether growth in the use of PD in the United States coincided with increases in the sizes of dialysis facility PD programs.^4^ They conducted a retrospective cohort study using publicly available data about dialysis facilities between 2010 and 2021 from the CMS Dialysis Facility Compare Website. Dialysis facility sizes were measured in three categories based on previous literature demonstrating an association between certain facility sizes and quality: 0 to 20 patients, 21 to 50 patients, and >50 patients. In multivariable mixed-effects regression models, the authors assessed for associations between calendar year and facility size categories.

The number of facilities providing PD increased from 2157 in 2010 to 3522 in 2021, indicating growth over time in the overall market for PD. While the proportion of facilities providing PD increased from 38.4% to 44.6%, this increase was largely due to increases in the proportion of dialysis facilities with larger sized PD programs. Between 2010 and 2021, the proportion of dialysis facilities that provided PD and that had between 20 and 50 patients increased from 22.9% to 29%. The proportion with >50 PD patients increased from 3.5% to 5.9%. Meanwhile, the proportion with <20 PD patients remained virtually unchanged during the study period. These trends were statistically significant in adjusted models. The authors conclude that growth in the number of facilities achieving important center effect thresholds suggests potential improvements in the quality of PD care delivery across the United States.

Up-front costs associated with opening new dialysis facilities may be one reason why growth in the proportion of smaller facilities was limited. For instance, a new dialysis program must incur costs associated with identifying a physician referral base, purchasing new equipment, training employees, and building processes and expertise for the delivery of PD. Compared with these startup costs, it may be less expensive to dialyze more patients at existing facilities. Economies of scale, where the cost per patient declines as the number of patients treated increases, may have also made the expansion of existing facilities a more financially attractive strategy. Such efficiencies have been observed in the broader dialysis setting and are likely applicable to PD as well.^5^

As the authors note, improvements in the quality of PD care are one possible consequence of growth in the proportion of larger dialysis facilities. However, an emphasis on larger center size may also carry drawbacks. First, by deterring providers from establishing new centers, a focus on increasing the size of existing centers could limit gains in access to care that might otherwise occur in the setting of market growth. Many patients prefer to travel shorter distances to receive dialysis. Although a preference for shorter travel distances is most pronounced in in-center hemodialysis, this preference has also been described in PD in instances when the nearest in-center hemodialysis facility is far from a patient's residence.^6^ If growing demand for PD is met by increases in the overall number of facilities, patients could benefit from increased accessibility and more localized care options. This would be especially impactful in areas previously underserved by dialysis services. If, instead, existing facilities accommodate increasing demand by becoming larger, patients may lose the opportunity to have more facilities closer to home. Similar concerns have been voiced about the implications of centralization in other areas of health care.^7^

Growth in the size of established providers could also make markets for PD less competitive than they would otherwise be. As markets grow, there is an opportunity for the entry of new providers to satisfy the increasing demand, increasing provider diversity and stimulating competition. Evidence in dialysis suggests that more competition among providers is associated with improved health outcomes.^8^ Moreover, market competition could help lower prices paid by private insurers. Yet, growth in the size of PD markets is less likely to lead to more competitive markets when demand is met by increasing sizes among existing providers, as was observed by Shah *et al*.

An emphasis on best practices within centers may be able to improve outcomes in the absence of achieving requisite size thresholds. For example, a retrospective cohort study of the Australia and New Zealand Dialysis and Transplant Registry examining the effect of center specific factors on peritonitis outcomes found that centers treating suspected peritonitis empirically with broad-spectrum antibiotics achieved higher cure rates.^9^ Another retrospective analysis demonstrated that the risk of peritonitis was influenced by factors such as the presence of a specialized PD nurse at the center and the performance of a home visit prior to PD initiation.^10^ Standardized protocols and targeted interventions such as these may help to improve outcomes, independent of facility size.

In summary, Shah *et al*. describe an increasing demand for PD in the United States that has been met by both an increase in the number of facilities providing PD as well as expansion of existing ones. This growth in facility size is somewhat encouraging, given its association with improved health outcomes. However, growth in the sizes of existing PD facilities may also have led to missed opportunities to improve access to and quality of care through the entry of new facilities into markets.

## References

[1] United States Renal Data System. 2024 USRDS Annual Data Report: Epidemiology of Kidney Disease in the United States. National Institutes of Health, National Institute of Diabetes and Digestive and Kidney Diseases; 2024.

[2] Koukounas KG Kim D Patzer RE, . Pay-for-performance incentives for home dialysis use and kidney transplant. JAMA Health Forum. 2024;5(6.9):e242055. doi:10.1001/jamahealthforum.2024.2055 38944762 PMC11215557

[3] Htay H Cho Y Pascoe EM, . Multicenter registry analysis of center characteristicsassociated with technique failure in patients on incident peritoneal dialysis. Clin J AmSoc Nephrol. 2017;12(7):1090–1099. doi:10.2215/CJN.12321216 PMC5498362 28637862

[4] Shah AD Ariff A Sammartino CJ Raker CA Hu SL. Scaling peritoneal dialysis: center effects thresholds in US facilities. Kidney360. 2026;7(1):150–156. doi:10.34067/KID.0000000969 40857112 PMC12889935

[5] Hirth RA Held PJ Orzol SM Dor A. Practice patterns, case mix, medicare payment policy, and dialysis facility costs. Health Serv Res. 1999;33(6):1567–1592. PMID: 10029498. 10029498 PMC1070337

[6] Pattharanitima P El Shamy O Chauhan K, . The association between prevalence of peritoneal dialysis versus hemodialysis and patients' distance to dialysis-providing facilities. Kidney360. 2021;2(12):1908–1916. PMID: 35419529. doi:10.34067/KID.0004762021 35419529 PMC8986048

[7] Huguet M. Centralization of care in high volume hospitals and inequalities in access to care. Soc Sci Med. 2020;260:113177. doi:10.1016/j.socscimed.2020.113177 32712556

[8] Saeed MK Ho V Erickson KF. Consolidation in dialysis markets-causes, consequences, and the role of policy. Semin Dial. 2020;33(1):90–99. doi:10.1111/sdi.12855 31930560

[9] Htay H Cho Y Pascoe EM, . Center effects and peritoneal dialysis peritonitis outcomes: analysis of a national registry. Am J Kidney Dis. 2018;71(6):814–821. doi:10.1053/j.ajkd.2017.10.017 29289475

[10] Béchade C Guillouët S Verger C Ficheux M Lanot A Lobbedez T. Centre characteristics associated with the risk of peritonitis in peritoneal dialysis: a hierarchical modelling approach based on the data of the French language peritoneal dialysis registry. Nephrol Dial Transplant. 2017;32(6):1018–1023. doi:10.1093/ndt/gfx051 28472525

